# Static and dynamic functional connectome reveals reconfiguration profiles of whole-brain network across cognitive states

**DOI:** 10.1162/netn_a_00314

**Published:** 2023-10-01

**Authors:** Heming Zhang, Chun Meng, Xin Di, Xiao Wu, Bharat Biswal

**Affiliations:** The Clinical Hospital of Chengdu Brain Science Institute, MOE Key Laboratory for Neuroinformation, Center for Information in Medicine, School of Life Science and Technology, University of Electronic Science and Technology of China, Chengdu, China; Department of Biomedical Engineering, New Jersey Institute of Technology, Newark, NJ, USA

**Keywords:** Network reconfiguration, Cognitive process, Drift diffusion model, Static functional connectivity, Dynamic functional connectivity

## Abstract

Assessment of functional connectivity (FC) has revealed a great deal of knowledge about the macroscale spatiotemporal organization of the brain network. Recent studies found task-versus-rest network reconfigurations were crucial for cognitive functioning. However, brain network reconfiguration remains unclear among different cognitive states, considering both aggregate and time-resolved FC profiles. The current study utilized static FC (sFC, i.e., long timescale aggregate FC) and sliding window–based dynamic FC (dFC, i.e., short timescale time-varying FC) approaches to investigate the similarity and alterations of edge weights and network topology at different cognitive loads, particularly their relationships with specific cognitive process. Both dFC/sFC networks showed subtle but significant reconfigurations that correlated with task performance. At higher cognitive load, brain network reconfiguration displayed increased functional integration in the sFC-based aggregate network, but faster and larger variability of modular reorganization in the dFC-based time-varying network, suggesting difficult tasks require more integrated and flexible network reconfigurations. Moreover, sFC-based network reconfigurations mainly linked with the sensorimotor and low-order cognitive processes, but dFC-based network reconfigurations mainly linked with the high-order cognitive process. Our findings suggest that reconfiguration profiles of sFC/dFC networks provide specific information about cognitive functioning, which could potentially be used to study brain function and disorders.

## Author Summary

A key question about functional brain connectome is whether a specific cognitive process depends on stronger or weaker network reconfiguration. A wide range of reconfiguration profiles based on both sFC and dFC should be taken into account to provide more detailed information. The current study first investigated static and dynamic network reconfigurations between three cognitive states and then linked them with behavioral parameters reflecting sensorimotor and cognitive processes. We further explored the relationship between sFC and dFC reconfiguration patterns. This work contributes to better understanding of relationships between cognitive functioning and network reconfiguration.

## INTRODUCTION

The whole-brain functional connectivity (FC), conceptualized as functional connectome, is closely linked with the manifestation of complex brain functions (Bullmore & Sporns, 2009). The convergent connectome architectures between rest and task states suggest that intrinsic and task-evoked connectomes depend on a general architecture of brain connectome (Gratton et al., 2018; Laumann & Snyder, 2021; Zuo et al., 2018). Therefore, FC networks of rest and task states are strongly related so that the task performance and cognitive functioning could be predicted by them (Cole et al., 2014; Greene et al., 2018). Emerging evidence supports the reconfiguration profiles of brain connectome between rest and task are also behaviorally relevant (Buckner et al., 2013; Mennes et al., 2013; Zuo et al., 2018), including network hubs shifted from primary to executive control network (Bolt et al., 2017), opposing changes for primary and fronto-parietal network (FPN) (Fransson et al., 2018), varied edge strength and more integrated network topology (Hearne et al., 2017), as well as greater network reconfiguration associated with faster information processing (Jung et al., 2018). Yet some studies suggest less task-related FC network reconfigurations are associated with higher intelligence or better cognitive performance (Schultz & Cole, 2016; Westphal et al., 2017; Zuo et al., 2018). Therefore, the relationships between FC network reconfigurations and specific cognitive process remain unclear. A recent study suggests there are different representations of cognitive processes in a hierarchical cortical gradient (Ito et al., 2020). There might exist separate and specific associations between network reconfigurations and low- and high-order functioning.

There are two FC approaches widely applied to study the overall and time-varying profiles of functional brain network (Betzel et al., 2016; Finc et al., 2020; Hutchison et al., 2013; Lurie et al., 2020). The aggregate FC over the timescales, which ignores temporal variations of FC, is estimated over the duration of whole cognitive state, namely static FC (sFC). The FC fluctuations over short timescales are often estimated based on within-state sliding windows, namely dynamic FC (dFC). While dFC delineates time-resolved properties of FC networks and sFC provides a long timescale aggregate description, they are partly related with each other and jointly provide more detailed information about functional systems (Betzel et al., 2016; Liégeois et al., 2020). The N-back task involves complex cognitive processes (Jaeggi et al., 2010), which can provide different level of cognitive challenges (Shine & Poldrack, 2018) or cognitive load states (Zuo et al., 2018). Previous research mainly studied the relationships between sFC/dFC network reconfigurations and task performance like accuracy and reaction time (Braun et al., 2015; Fong et al., 2019; Schultz & Cole, 2016; Zuo et al., 2018). The drift diffusion model enables one to separate and delineate sensorimotor and cognitive process (Wagenmakers et al., 2007). Recent studies reported that network reconfigurations during cognitively challenging task were linked with sensorimotor and cognitive process using sFC and dFC approach separately (Fransson et al., 2018; Shine et al., 2016; Shine & Poldrack, 2018). As FC network reconfigurations have shown a wide range of profiles, including edge weights and topological properties, it is necessary to study comprehensive reconfiguration profiles based on short and long timescale dynamic interaction by combining sFC and dFC approaches. According to previous literature, we hypothesized that sFC and dFC together may reveal more detailed and specific reconfiguration profiles to underpin cognitive functioning. Meanwhile, considering that sFC and dFC are related to each other in resting state (Betzel et al., 2016; Liégeois et al., 2020; Zhang et al., 2018), there might exist correlated reconfiguration profiles between sFC and dFC, which still remains to be elucidated.

To address these questions, the current study combined sFC and dFC approaches to investigate the network reconfiguration profiles from rest to cognitively challenging task states, concerning edge weight and network topology. According to the cognitive load theory (Christophel et al., 2017; Jenkins, 2019), as well as previous studies (Braun et al., 2015; Liang et al., 2016; Shine et al., 2016; Zhang et al., 2022b), the current study defined three cognitive states by the rest and 1- and 2-back task states, which were associated with increasing cognitive demand. Recent work reported task- or learning-related reconfigurations in network topology such as enhanced functional integration for FPN (Finc et al., 2020; Fransson et al., 2018). The FPN is the core network for adaptive task control, which responds to multiple demands (Duncan et al., 2012), underpins cognitive flexibility (Cole et al., 2013), and reconfigures possibly more than other networks to achieve better task performance (Braun et al., 2015; Cocuzza et al., 2020; Murphy et al., 2020), including cognitive processes reflected by the drift diffusion model (Fransson et al., 2018). Therefore, FPN was selected to further study the association between reconfiguration profiles and cognitive process. Last but not least, the current study explored the relationship between reconfigurations of sFC and dFC.

## MATERIALS AND METHODS

### Participants and Study Procedure

Fifty young healthy participants (22 ± 1.3 years old; 24 males; all right-handed) from the University of Electronic Science and Technology of China undertook this study after providing informed written consent. The current study was approved by the local Ethics Committee of the university. The details about participants, exclusion criteria, continuous N-back task design, resting- and task-state fMRI data collection can be found in our prior study (Zhang et al., 2022b). Briefly, each participant underwent multiple fMRI scans of eyes-open resting-state, working memory 1-back and 2-back continuous task (Krienen et al., 2014). In total, three cognitive load states were characterized by increasing cognitive challenge from rest, 1-back to 2-back (Zhang et al., 2022b), and used to investigate whole-brain FC network reconfigurations in the current study.

### Drift Diffusion Model and Cognitive Processes

For the cognitively challenging task, the drift diffusion model provides a decomposition of behavioral performance (response time and accuracy) into cognitively relevant latent variables, representing the speed and accuracy of information processing (drift rate), the speed of perceptual and motor processes (nondecision time), and the flexibility of response caution (boundary separation or so called decision threshold) (Voss et al., 2013; Wagenmakers et al., 2007). Specifically, the decision threshold is related to decision-making conservativeness. For example, a wider decision threshold requires more information before a decision can be made (more accurate but slower responses), whereas narrower threshold leads to faster but more error-prone decisions. A higher drift rate suggests faster and more accurate decisions. So drift rate is directly related to decision process, and may infer individual study ability or task difficulty (Wagenmakers et al., 2007). In contrast, nondecision time is not directly related to the decision process (Wagenmakers et al., 2007), but indicates the time of sensorimotor process (Fransson et al., 2018; Shine et al., 2016), as well as stimulus encoding (Pedersen et al., 2017). Since decision process is a higher order cognitive process than sensorimotor and stimulus encoding (Wang & Chiew, 2010), drift rate and decision threshold indicates high-order cognitive process, while nondecision time indicates sensorimotor process and low-order cognitive process.

### fMRI Preprocessing and Network Construction

The resting-state and task fMRI data were preprocessed the same way as in our prior study (Zhang et al., 2022b). No participant was excluded according to the excessive motion criteria (maximal cumulative head motion of 2 mm or 2 degrees, or mean frame-wise displacement (FD) of 0.2 mm) (Di et al., 2020). For quality control, we examined and found that none of correlations between subsequent FC measures and mean FD was significant (FDR-corrected *p* > 0.05). Recent literature suggested moderate rather than aggressive motion denoising approach such as ICA-based motion denoising, especially for task fMRI (Caballero-Gaudes & Reynolds, 2017; De Blasi et al., 2020; Mayer et al., 2019). Therefore, the current study employed nuisance variable regression for both resting-state and task fMRI data, including 24 head motion time courses, average signals of cerebrospinal fluid and white matter. Additional preprocessing steps of linear detrend, 8-mm smoothing, and band-pass filtering of 0.008–0.25 Hz were conducted before FC network analyses (Finc et al., 2020).

In the current study, whole-brain functional networks were constructed based on 286 nodes defined by coordinate-based spherical regions of interest, which are attributed to 14 functional modules (Brandl et al., 2018; Power et al., 2011) (see Figure 2A). The edges were defined by pairwise Pearson correlation between nodal time series followed by Fisher’s *z* transformation, resulting in multiple 286 × 286 FC matrices for each participant and each cognitive state. Here, both sFC and dFC were utilized to study the network reconfiguration. For dFC, the sliding window approach was used to compute dFC with the window length of 40 s and step of 20 s according to recent study (He et al., 2018). Alternative window lengths and steps were tested in validation analyses. To keep the same number of windows for resting and task states, the first and last 50 time points in resting state were discarded, which resulted in 14 windows for each cognitive state individually.

### sFC Network Reconfiguration

The average sFC network was obtained for the standard architecture of functional brain organization (Cole et al., 2014) and to quantify the extent of sFC network similarity between cognitive states. Pearson correlations based on edge weights were computed by using vectorized sFC matrices. Then, graph theoretic analysis was conducted for the properties of global and modular network topology and later to compare between cognitive states.

To evaluate topological organization, sFC networks were first binarized (except for modularity) by using the density threshold of 20% combined with the minimum spanning tree, in order to ensure small-world and fully connected brain network (Morgan et al., 2018; Termenon et al., 2016). Then we calculated graph metrics including small-worldness (sigma), global efficiency, clustering coefficient, and betweenness centrality (Rubinov & Sporns, 2010). Small-world networks often have sigma ≫ 1 and should be simultaneously highly segregated and integrated compared to random networks, for example, having high scores of clustering coefficient and global efficiency (Rubinov & Sporns, 2010; Watts & Strogatz, 1998). Betweenness centrality can reflect the average extent of importance of nodes or edges for overall functional integration (Rubinov & Sporns, 2010). Additionally, the modularity Q quantifies the density of connections within clusters compared to the density of connections between clusters (Blondel et al., 2008). Higher modularity is indicated by stronger within-module connections and weaker between-module connections (Rubinov & Sporns, 2010). In the current study, Q was computed by using the Louvain modularity algorithm implemented in the Brain Connectivity Toolbox (Rubinov & Sporns, 2010, 2011). In line with prior works (Jung et al., 2018; Zuo et al., 2018), we used 100 repetitions to identify the maximal Q and community structure:

$$\text{Q}=\frac{1}{2m}\sum_{ij}\left[A_{ij}-\gamma \frac{k_{i}k_{j}}{2m}\right]\;\delta \left(c_{i},c_{j}\right)$$

where *m* is the total edge weight of the network, *A*_*ij*_ represents the weight of the edge between *i* and *j*, *c*_*i*_, and *c*_*j*_ are the assignment of the node *i* and *j* to a community, and *γ* is the structural resolution parameter. We used the default *γ* of 1 for all analyses in line with prior work (Bassett et al., 2011; Zhou et al., 2022). *k*_*i*_ and *k*_*j*_ are the weighted degrees of nodes *i* and *j*. The Kronecker delta function *δ*(*c*_*i*_, *c*_*j*_) equals 1 if nodes *i* and *j* belong to the same module, and equals 0 otherwise. To display the modular structure and its reconfigurations, we also computed the modularity at the group level based on the averaged sFC network of each state.

To compare the extent of functional integration at the modular level between states, modular segregation index (MSI) was computed for each subject, cognitive state, and a priori module (segregation/integration). A negative value of MSI suggests stronger functional integration. (Chan et al., 2014; Ma et al., 2020). Here 12 a priori modules were considered since the function of cerebellum and uncertain modules is complex (Clark et al., 2021; Power et al., 2011; Stoodley & Schmahmann, 2010):

$${\text{MSI}}_{i}=\frac{E_{\text{intra}}-E_{\text{inter}}}{E_{\text{intra}}}$$

where *E*_*intra*_ is the number of intranetwork edges of module *i*, and *E*_*inter*_ is the number of internetwork edges between-module *i* and all other modules. The sFC network reconfiguration was analyzed by overall profiles (similarity and average network modular structure) and statistical comparisons of global and modular graph metrics between cognitive states.

### dFC Network Reconfiguration

The dFC network reconfigurations were analyzed mainly at the modular level, by the alteration of dynamic MSI (dMSI, same method with above and computed for each window), as well as the between-state similarity of allegiance matrices. Notably, the allegiance matrix provides quantitative information about the dynamic association between nodes and modules during resting or task state, which take all positive edge weights into account (Finc et al., 2020). Given a network allegiance matrix *P*, *P*_*ij*_ represents the fraction of network layers for which node *i* and node *j* belong to the same module:

$$P_{i,j}=\frac{1}{rw}\sum_{r=1}^{r}\sum_{w=1}^{w}a_{i,j}$$

where *r* is the number of repetitions of the multilayer community detection algorithm (here, *r* = 100), and *w* is the number of windows (here, *w* = 14). If nodes *i* and *j* are in the same module, *a*_*i*,*j*_ is 1, otherwise *a*_*i*,*j*_ is 0. Pearson correlations between the allegiance matrices of rest, 1-back and 2-back states were computed to evaluate the similarity of dFC network organization across cognitive states. On the other hand, dMSI enables us to compare individual modular reconfiguration between sFC and dFC, concerning the extent of functional integration over different timescales. Here, based on 14 dFC matrices, we computed the standard deviation of dMSI (*SD*, reflecting the fluctuating extent), and the speed of dMSI (i.e., mean absolute value of the first derivative of dMSI). The higher *SD* and speed of dMSI indicated larger and faster variability in the dynamic modular network organization.

### Statistical and Correlation Analyses

For behavioral performance reflecting cognitive process, the paired *t* test was utilized to determine the influence of cognitive state on parameters derived from the drift diffusion model. For sFC/dFC network reconfigurations, repeated-measure one-way ANOVA was used to determine the effect of cognitive state on FC measures. The false discovery rate (FDR) was employed to correct for multiple comparisons. A post hoc *t* test was conducted following significant main effects. Then the relationships between global network reconfigurations and parameters of drift diffusion model were evaluated. For modular reconfigurations, since FPN is the core network substrating working memory and adaptive task control (Cole et al., 2013; Sherwood et al., 2016), we tested if FPN reconfigurations correlated with parameters of the drift diffusion model by using SPSS 22 (IBM, Armonk, NY). Last but not least, the correlations between sFC and dFC reconfigurations were explored.

### Validation Analyses

First, previous literature reported the impact of micromovement on FC measures (Ciric et al., 2017; Power et al., 2012) as well as potential correlations between head motion and behavioral variables (Siegel et al., 2017). Therefore, ANOVA and brain-behavioral correlation results were verified between without and with regressing out mean FD value from FC and reconfiguration measures to test the reliability of results. For network similarity, mean FD values of two states and their interaction were regressed out. Second, in the current study, we computed the results of modularity by using the modularity maximization method for community detection following previous studies (Bassett et al., 2013; Jung et al., 2018; Zuo et al., 2018). Another method used to find a single solution for the nonconvex modularity maximization is consensus clustering, which generates an agreement matrix for determining a representative network partition from multiple subjects or from a set of temporal dynamic networks (Kabbara et al., 2019; Rasero et al., 2017). Therefore, we also verified the main findings in the current study by using the consensus clustering (Lancichinetti & Fortunato, 2012). For details, see the Supporting Information. Third, validation analyses were conducted to test if binarized sFC network results were sensitive to different density thresholds (15%, 20%, and 25%). Furthermore, dFC results were validated by considering alternative window lengths (30 s, 40 s, and 50 s) and step lengths (10 s, 20 s, and 30 s).

## RESULTS

### Behavioral Changes for Cognitive Processes

**Figure 1.**
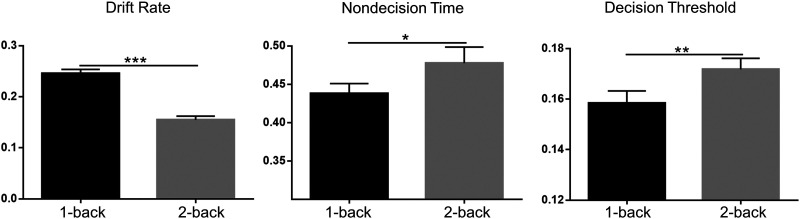
Behavioral parameters varied with the cognitive load. The significant results from paired *t* test were indicated by *p ≤ 0.05; **p ≤ 0.01; ***p ≤ 0.001. Error bars indicated the standard error.

The results of paired *t* test demonstrated significant behavioral differences related to sensorimotor and cognitive processes (Figure 1). For example, see lower drift rate (*t*_(49)_ = 11.20, *p* < 0.001, Cohen’s *d* = 0.1869), higher nondecision time (*t*_(49)_ = −2.28, *p* = 0.0272, Cohen’s *d* = 0.1864), and higher decision threshold (*t*_(49)_ = −3.33, *p* = 0.0016, Cohen’s *d* = 0.1028), in the 2-back than 1-back state. It may suggest that, at higher cognitively challenging state, participants have slower sensorimotor and low-order cognitive processing, as well as slower and more conservative high-order cognitive processing, for making correct decisions (Wagenmakers et al., 2007).

### Reconfiguration Profiles of sFC Network

**Figure 2.**
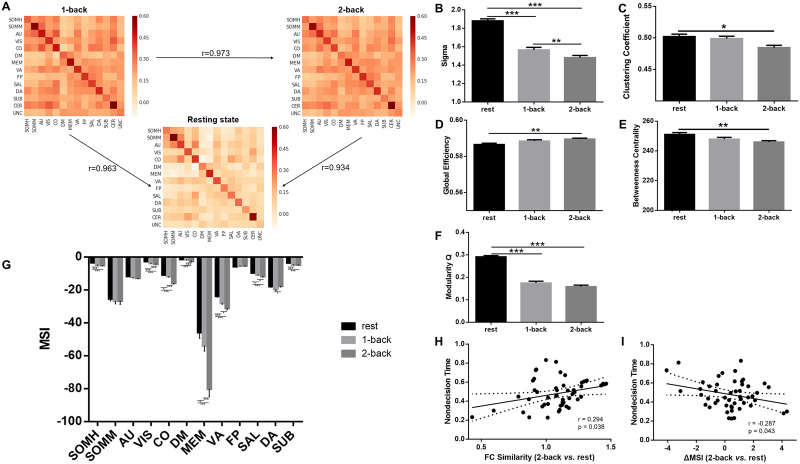
SFC network reconfiguration results. (A) Similar FC edge weights between cognitive states. (B–G) ANOVA and post hoc paired *t* test results for global and modular network topology. The significant results were indicated by ***p ≤ 0.001, **p ≤ 0.01, *p ≤ 0.05, FDR-corrected. Error bars indicate standard error. (H–I) Associations between sFC and FPN reconfiguration and parameters of drift diffusion model. SOMH = somatomotor hand; SOMM = somatomotor mouth; AU = auditory; VIS = visual; CO = cingulo-opercular; DM = default mode; MEM = memory; VA = ventral attention; FP = fronto-parietal; SAL = salience; DA = dorsal attention; SUB = subcortical; CER = cerebellum; UNC = uncertain; MSI = modular segregation index.

The results of sFC network similarity showed very high correlation in edge weights between states (Figure 2A; in detail, 1-back vs. rest: *r* = 0.963, *p* < 0.00001; 2-back vs. rest: *r* = 0.934, *p* < 0.00001; 1-back vs. 2-back: *r* = 0.973, *p* < 0.00001), together with preserved property of small-world network (sigma > 1.4), suggesting overall stable architecture in functional connectome across the cognitive states. However, we also found that, network similarity between rest and 2-back state was positively correlated with the nondecision time of 2-back state (*r* = 0.294, *p* = 0.038; Figure 2H). It might be due to subtle FC changes or topological reorganization so that participants having larger reconfiguration from standard connectome architecture (lower network similarity) could have faster sensorimotor and low-order cognitive processes.

Results of graph analyses and ANOVA demonstrated significant main effects of cognitive state on global and modular network topology (FDR-corrected *p* < 0.05), suggesting pronounced reconfigurations (Table 1). Post hoc comparisons revealed a consistent trend that the higher cognitive load the stronger functional integration, based on global efficiency and clustering coefficient in overall sFC network, as well as MSI in somatomotor hand (SOMH), visual (VIS), cingulo-opercular (CO), default mode (DM), memory (MEM), ventral attention (VA), salience (SAL), and subcortical (SUB) (Figure 2B–G and Table 1). Besides, a decreasing trend displayed for sigma, betweenness centrality, and modularity, suggesting altered global network topology from rest to cognitively challenging task. Meanwhile, dorsal attention network showed inconsistent trend of reconfigurations that were different from other eight modules with significant results of main effect (Figure 2G). In detail, as the cognitive load increased, sigma reduced (FDR-corrected *p* < 0.05, Cohen’s *d* ranging from 0.4641 to 2.5770; Figure 2B), clustering coefficient reduced (FDR-corrected *p* < 0.05, Cohen’s *d* = 0.7562; Figure 2C), global efficiency increased (FDR-corrected *p* < 0.05, Cohen’s *d* = 0.0716; Figure 2D), betweenness coefficient reduced (FDR-corrected *p* < 0.05, Cohen’s *d* = 0.0751; Figure 2E), and modularity reduced (FDR-corrected *p* < 0.05, Cohen’s *d* ranging from 0.9314 to 0.5422; Figure 2F), but none of their reconfigurations was correlated with drift rate or nondecision time (*p* > 0.05).

**Table 1.** Statistics of the global and modular topology by ANOVA

	**Effect of cognitive state**		
	***F*_2, 104_**	***P* value**	***η*_p_^2^**
**Global Topology**			
Sigma	108.013	<0.001	0.688
Clustering coefficient	4.664	0.030	0.082
Global efficiency	6.193	0.005	0.112
Betweenness coefficient	6.007	0.005	0.109
Modularity	182.344	<0.001	0.788
**Modular topology**			
MSI of SOMH	31.741	<0.0001	0.393
MSI of VIS	54.490	<0.0001	0.527
MSI of CO	42.739	<0.0001	0.466
MSI of DM	60.849	<0.0001	0.554
MSI of MEM	36.995	<0.0001	0.430
MSI of VA	21.845	<0.0001	0.308
MSI of SAL	11.324	<0.0001	0.188
MSI of DA	4.010	0.025	0.076
MSI of SUB	33.879	<0.0001	0.409

*Note*. MSI = modular segregation index; SOMH = somatomotor hand; VIS = visual; CO = cingulo-opercular; DM = default mode; MEM = memory; VA = ventral attention; SAL = salience; DA = dorsal attention; SUB = subcortical.

**Figure 3.**
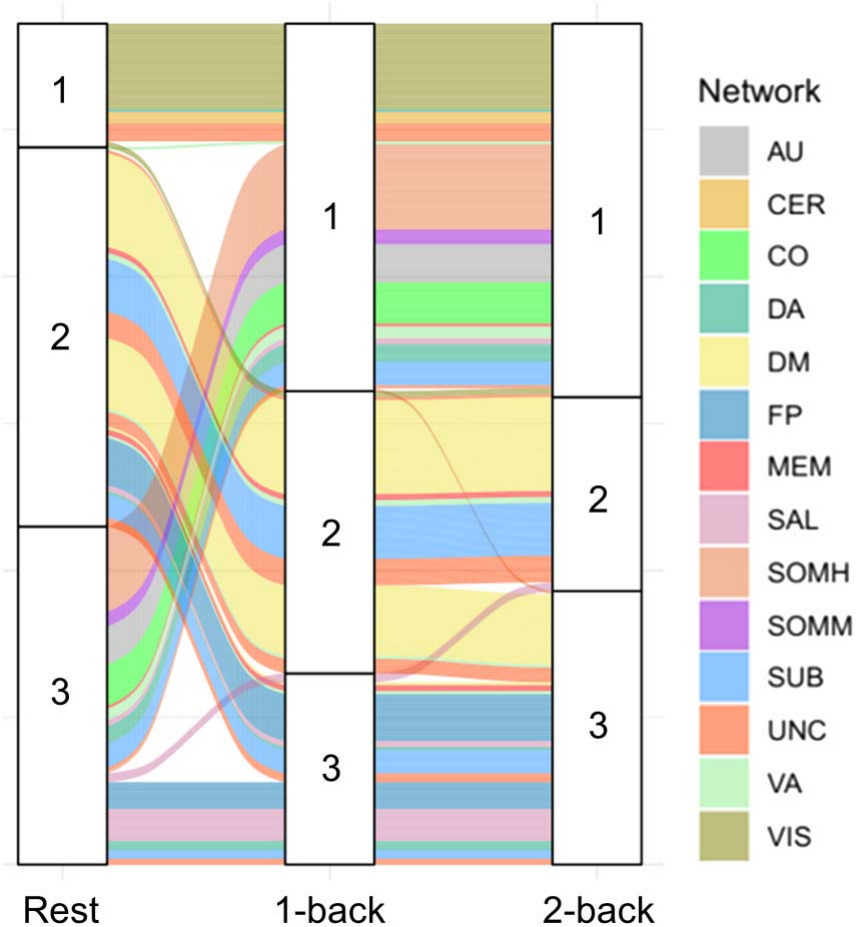
The alluvial flow plot demonstrated more reconfigurations in the modular structure from rest to 1-back than from 1-back to 2-back. Each streamline represents a node in the network, colored by the affiliation to a priori modules. Using default parameter for Louvain modularity maximization, three modules were consistently identified across three states. From 1-back to 2-back task state, the modular structure was largely stable except some networks in module 2 and 3, like DM and SAL, were subdivided and switched to other modules. From rest to task state, module 3 separated a part to join in enlarged module 1, mainly involving SOMM, SOMH, AU, CO, DA, and VA networks, while module 2 gave a part to module 3, mainly involving FP, SUB, and SAL networks.

Results of modular structure displayed more reconfigurations from rest to 1-back than from 1-back to 2-back (Figure 3). From 1-back to 2-back task state, the modular structure was largely stable, though some parts of DM were reconfigured to be together with fronto-parietal (FP) and salience (SAL) networks. From rest to task state, widespread reconfigurations were present with regard to primary (such as SOMH, AU) and association cortex (such as FP, SAL). Interestingly, FPN (i.e., FP in Figure 3) seemed to be more integrated from rest to task states by merging into one module, but MSI of FPN was not significant concerning main effect of cognitive state (FDR-corrected *p* > 0.05; Figure 2G). However, MSI difference of 2-back versus rest in FPN showed significant negative correlation with nondecision time of 2-back state (*r* = −0.287, *p* = 0.043; Figure 2I), suggesting participants with enhanced segregation in FPN (higher MSI of FPN in 2-back than rest) at cognitively challenging task state have faster sensorimotor and low-order processes.

### Reconfiguration Profiles of dFC Network

**Figure 4.**
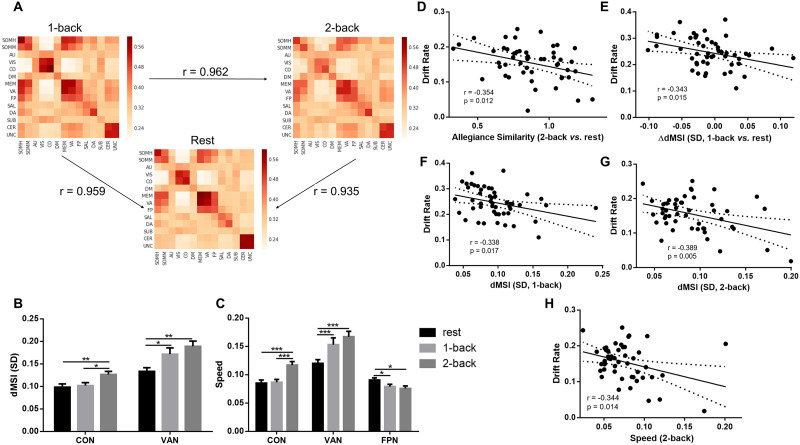
dFC network reconfiguration results. (A) Similar allegiance matrix between cognitive states. (B and C) ANOVA and post hoc paired *t* test results for the *SD* and speed of dMSI. The significant results were indicated by ***p ≤ 0.001, **p ≤ 0.01, *p ≤ 0.05, FDR-corrected. Error bars indicate standard error. (D–H) Associations between dFC and FPN reconfiguration and parameters of drift diffusion model. CON = cingulo-opercular network; VAN = ventral attention network; dMSI = dynamic modular segregation index; *SD* = standard deviation.

The result of allegiance matrices similarity showed very high correlation in modular variability between states (Figure 4A; in detail, 1-back vs. rest: *r* = 0.959, *p* < 0.00001; 2-back vs. rest: *r* = 0.935, *p* < 0.00001; 1-back vs. 2-back: *r* = 0.962, *p* < 0.00001), suggesting consistent modular reconfiguration pattern with some subtle changes across the cognitive states. However, we also found that allegiance matrices similarity between rest and 2-back state was negatively correlated with the drift rate of 2-back state (*r* = −0.354, *p* = 0.012; Figure 4D). It might be due to subtle FC changes or topological reorganization so that participants having larger variability of the connectome architecture (lower similarity) could have faster speed and higher accuracy of information processing.

Results of graph analyses and ANOVA demonstrated significant main effects of cognitive state on dMSI properties (FDR-corrected *p* < 0.05), suggesting pronounced faster and larger variability of dFC (Table 2). Post hoc comparisons revealed a consistent trend that for cingulo-opercular network (CON) and ventral attention network (VAN), the higher cognitive load the faster and larger dFC variability (Figure 4B and C and Table 2). However, the FPN showed the opposite trend that the higher cognitive load the slower dFC variability (Figure 4C and Table 2). In detail, as the cognitive load increased, the dynamic CON and VAN variability increased (CON: Cohen’s *d* ranging from 0.1129 to 0.4916; VAN: Cohen’s *d* ranging from 0.2186 to 1.2393; Figure 4B), while the speed of dynamic CON and VAN increased (CON: Cohen’s *d* ranging from 0.1795 to 0.7744; VAN: Cohen’s *d* ranging from 0.1362 to 0.4947; Figure 4C) However, the FPN showed a uniformly decreasing trend as the cognitive load increased (Cohen’s *d* = 0.1917; Figure 4C).

**Table 2.** Statistics of the *SD* and the speed of the dMSI by ANOVA

	**Effect of cognitive state**		
	***F*_2, 104_**	***P* value**	***η*_p_^2^**
**SD of dMSI**			
CO	5.837	0.004	0.106
VA	7.501	0.001	0.133
**Speed of dMSI**			
CO	12.507	<0.001	0.203
VA	7.745	<0.001	0.136
FP	5.779	0.004	0.105

*Note*. *SD*: standard deviation; dMSI, dynamic modular segregation index.

The correlation results revealed significant negative correlation between the change of dMSI (1-back minus rest) and drift rate of 1-back state (*r* = −0.343, *p* = 0.015; Figure 4E), suggesting participants with smaller dFC variability in FPN (lower SD of dMSI of FPN in 1-back than rest) have faster speed and higher accuracy of information processing. Moreover, the negative correlations between dMSI and drift rate were significant (*SD*: 1-back: *r* = −0.338, *p* = 0.017, Figure 4F; 2-back: *r* = −0.380, *p* = 0.005, Figure 4G; speed: 2-back: *r* = −0.344, *p* = 0.014, Figure 4H). The results indicated that participants with smaller dFC variability in FPN during both the simple and challenging cognitive task state corresponded to faster speed and higher accuracy of information processing.

### Correlations Between sFC and dFC Network Reconfiguration Properties

**Figure 5.**
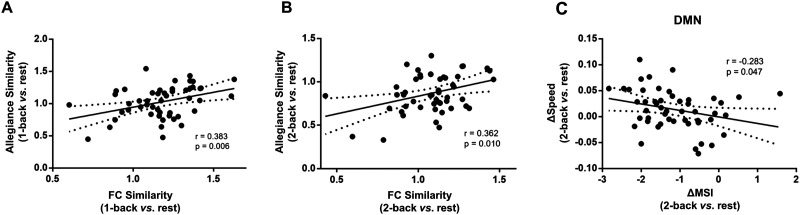
Associations between sFC and dFC network reconfigurations by using Pearson correlation. (A and B) The sFC and dFC reconfigurations showed positive correlation in task states. (C) Compared with resting state, the larger variability of the network showed higher network integration in 2-back state in DMN.

To investigate the relationship between the two aspects of sFC and dFC network reconfiguration modulated by cognitive loads, as well as the relationship between network similarity, allegiance matrix similarity, MSI, and *SD* and speed of dMSI were explored in the current study. For the whole-brain level, the results showed significant positive correlation between network similarity and allegiance matrix similarity (1-back: *r* = 0.383, *p* = 0.006, Figure 5A; 2-back: *r* = 0.362, *p* = 0.010, Figure 5B). The results indicated that the sFC and dFC reconfiguration properties had consistent results in task states. For the modular level, the results showed significant negative correlation between the change of MSI and the speed of dMSI in default mode network (DMN) (2-back-rest: *r* = −0.283, *p* = 0.047, Figure 5C). The results indicated that compared with resting state, the larger variability and flexibility of the DMN showed higher network integration in 2-back state.

### Validation Results

Generally, the effects of cognitive states and correlation results remained largely unchanged in the validation analysis without and with regressing out mean FD. The results showed that no matter whether we regressed out head motion or not, the effects of cognitive states in sFC/dFC network properties consistently existed. Moreover, the trends of changes in each of sFC/dFC network properties are consistent between with- and without-mean FD regression (Supporting Information Table S1 and Figure S1). The correlation results showed consistently significant correlations between before and after head motion regress (Figure S2). In sum, the validation results suggested that our findings were largely unaffected by individual differences in head motion. The validation of modular structure reconfiguration showed similar results by using the modularity maximization method and consensus clustering method, except that at rest, the module 1 of “modularity maximization” was separated into two modules of “consensus clustering” (Figure S3). Our main results (20%) were largely reproducible at different density thresholds, and showed similar trends modulated by cognitive loads at different thresholds (Table S2 and S3; Figure S4 and S5). Besides, our main results (Figure 4) were largely insensitive to window length changes or step changes; although, the results of *SD* of dMSI in FPN as well as speed of dMSI in SAL, SOMH and SUB were altered (Figure S6).

## DISCUSSION

The current study investigated reconfigurations in static and dynamic functional connectome across cognitive states concerning network edge weight and topological organization. First, results demonstrated relatively stable connectome architecture concerning edge weights and node–module allegiances. Second, significant sFC and dFC changes in edge weight similarity, global and modular network topology provided more detailed and specific information about FC network reconfiguration from rest to task states. For FPN, the sFC network reconfiguration mainly corresponded to the sensorimotor and low-order cognitive processes, while the dFC network reconfiguration mainly corresponded to the high-order cognitive process. At last, correlation was found between sFC and dFC network reconfigurations. In sum, the current study has provided more comprehensive mapping for network reconfiguration profiles from rest to cognitively challenging task states, which could be useful for future study on brain function and disorders.

The sFC and dFC network reconfiguration properties were positively correlated. It confirms the consistent pattern of sFC and dFC in the task-versus-rest network reconfiguration in line with the literature. For example, Fong et al. (2019) reported that both sFC and dFC can predict individual differences in attention. Moreover, the sFC and dFC network reconfiguration properties can reflect the different aspects of individual cognitive processes. Specifically, the sFC network reconfiguration property had significant correlation with nondecision time, but the dFC network reconfiguration property had significant correlation with drift rate (Figures 2 and 4). As we already know, nondecision time is related to sensorimotor and low-order cognitive processes like stimulus encoding, while drift rate is critical for high-order cognitive process directly related to decision process (Wagenmakers et al., 2007). Some of previous literature reported that greater network reconfiguration corresponds to higher accuracy (Cohen & D’Esposito, 2016) and shorter reaction time (Zuo et al., 2018). There was a different view that smaller changes in connectome architecture between rest and task correspond to higher individual performance (Schultz & Cole, 2016). These inconsistent findings may be attributed to different methods used in the task-state FC assessment, like concatenating task blocks without or with regressing out task activity (Cohen & D’Esposito, 2016; Zuo et al., 2018), or directly using residuals from task regression (Schultz & Cole, 2016). The continuous design is simple but effective to characterize task-state FC (Krienen et al., 2014; Zhang et al., 2022a, 2022b) and avoids the methodological choice between task regression and block concatenation. Importantly, current findings based on the N-back task with continuous design provide the separate evidence between network reconfigurations and specific cognitive process.

Both sFC and dFC revealed relatively less reconfiguration in FPN than other functional networks across different cognitive states. Participants with stronger segregation in FPN in task state compared to resting state had shorter nondecision time, while participants with smaller variability of dynamic modular segregation in FPN had higher drift rate. Our results confirmed that FPN is an important network in cognitive process, associated with cognitive process such as attentional control (Mostofsky et al., 2009; Sherwood et al., 2016) and memory maintenance (D’Esposito et al., 2000; Gazzaley et al., 2004). Previous studies also reported functional integration in FPN that showed positive correlation with accuracy (Braun et al., 2015; Hearne et al., 2017) as well as drift rate (Fransson et al., 2018). The current study has provided novel evidence for FPN, which is associated with not only drift rate reflecting high-order cognitive process but nondecision time reflecting low-order cognitive and sensorimotor processes. Moreover, the overall network patterns for both sFC and dFC are largely reserved across different cognitive states, in terms of very high similarity in the sFC edge weights and in the allegiance weights of dynamic modular reconfiguration, which are correlated with nondecision time and drift rate. In sum, associations between sFC/dFC reconfiguration profiles and cognitive process are consistent for FPN and overall network patterns.

As for network topology, significant main effect of cognitive state on global efficiency and clustering coefficient suggested that as cognitive load increased, whole-brain integration increased consistently. This is in line with prior work showing that increased network integration links with improved task performance (Cohen & D’Esposito, 2016; Westphal et al., 2017). However, except for sigma, the difference of global network topology between 1- and 2-back was not significant. It may be because the integration is pronouncedly reconfigured from rest to task, but slightly varied when cognitive load changes (Bolt et al., 2017; Hearne et al., 2017; Kaposzta et al., 2021). On the other hand, the modular organization provided more information about sFC and dFC reconfigurations. Whole-brain network was consistently subdivided into three modules but reconfigured at each state (Figure 3). Results displayed more reconfigurations from rest to 1-back than from 1-back to 2-back, which is consistent with FC similarity results. The modules showed similar patterns with prior work (Hearne et al., 2017). Inside, module 1 mainly had visual network at rest, plus more sensory and motor networks at task. Module 2 mainly involved DMN as the task negative network (Fox et al., 2005). Module 3 seemed to have more integrated FPN and salience network at task than rest, and played critical roles in the cognitive process (Finn et al., 2019; Myers et al., 2017; Taghia et al., 2018). Meanwhile, part of DMN switched from module 2 to module 3 that involved FPN, probably because higher integration of FPN and DMN can facilitate cognitive process (Westphal et al., 2017). Using MSI and dMSI, the current study also revealed significant main effect of cognitive state on a priori modules, which extended previous evidence on increased integration within and between modules affected by cognitive load (Liang et al., 2016).

Last but not the least, validation analyses have shown that the main findings in the current study are largely reproducible considering several methodological issues. Besides, there exist some debates about path length defined by correlation-based FC, which is less intuitive than anatomical connections when charactering brain network topology. On the one hand, Pearson correlation cannot distinguish the direct and indirect influence in the functional network (Faskowitz et al., 2022; Liégeois et al., 2020; Sanchez-Romero & Cole, 2021). On the other hand, correlation-based FC reflects the information about neurobiological coupling and has contributed greatly to the brain network research (Faskowitz et al., 2022). Recent work suggests FC is constrained by underlying structure (such as gray matter covariance and white matter fiber connection) (Sun et al., 2022). Therefore, the results of path length (e.g., global efficiency, small-worldness, and betweenness centrality) in the current study should be interpreted as findings based on correlation-based FC network topology. Conceptual differences must be emphasized especially when comparing with results of structural brain network.

In conclusion, both sFC and dFC network reconfigurations substrate brain functioning changes underlying cognitive process, while sFC and dFC approaches can reflect shared and separate cognitive processes. Therefore, it is important to comprehensively study sFC and dFC reconfiguration profiles for better understanding cognitive state and brain function.

## ACKNOWLEDGMENTS

We thank all the participants of this study.

## SUPPORTING INFORMATION

Supporting information for this article is available at https://doi.org/10.1162/netn_a_00314.

## AUTHOR CONTRIBUTIONS

Heming Zhang: Conceptualization; Data curation; Formal analysis; Investigation; Methodology; Project administration; Software; Validation; Visualization; Writing – original draft; Writing – review & editing. Chun Meng: Conceptualization; Methodology; Supervision; Writing – review & editing. Xin Di: Conceptualization; Writing – review & editing. Xiao Wu: Writing – review & editing. Biswal Bharat: Resources; Supervision; Writing – review & editing.

## FUNDING INFORMATION

Chun Meng, National Natural Science Foundation of China (http://dx.doi.org/10.13039/501100001809), Award ID: 61871420, 62071109. Chun Meng, Natural Science Foundation of Sichuan Province (http://dx.doi.org/10.13039/501100018542), Award ID: 2022NSFSC0504.

## TECHNICAL TERMS

Functional connectivity: Statistical dependency of signals between brain regions, like Pearson correlation, can infer the functional interaction among distributed processes.
Network reconfiguration: Altered network patterns and topological properties between different cognitive states.
Cognitive state: A relatively long run with stable cognitive load for participants.
Static functional connectivity: The aggregate measure of functional connectivity over the timescales, computed based on the whole scan.
Dynamic functional connectivity: The temporal variants which can be computed using sliding windows, representing the transient functional connectivity over short timescales.
Drift rate: The speed of accumulation of decision-dependent evidence during decision-making, which is associated with high-order cognitive process.
Nondecision time: The time of process which is not directly related to the decision process, including sensorimotor process and stimulus encoding.
Module: A subnetwork that have the nodes more strongly connected with each other than to the rest of the system.
Segregation/integration: Number of connections occur primarily within or across functional networks.
