# Supplementary material for: Static and dynamic functional connectome reveals reconfiguration profiles of whole-brain network across cognitive states
Source: Netw Neurosci. 2023 Oct 1;7(3):1034–50. doi: 10.1162/netn_a_00314 (PMC10473282; doi:10.1162/netn_a_00314)
Supplement: Supplementary file 1 [file netn-7-3-1034-s001.pdf]

Title

Static and dynamic functional connectome reveals reconfiguration profiles of whole brain network across cognitive states

Author

Heming Zhang[1], Chun Meng[1✉], Xin Di[2], Xiao Wu[1], Bharat Biswal[1, 2✉]

[1] The Clinical Hospital of Chengdu Brain Science Institute, MOE Key Laboratory for Neuroinformation, Center for Information in Medicine, School of Life Science and Technology, University of Electronic Science and Technology of China, Chengdu 611731, China.

[2] Department of Biomedical Engineering, New Jersey Institute of Technology, Newark, NJ, 07102, USA.

Supplement Material

To examine if our results were affected by head motion, we regressed out mean frame-wise displacement (FD) from measures before ANOVA and correlation analyses in the current study. We found that, significant results reported in the current study were all reproducible (SI Table 1 and SI Figure 1-2), with slightly different p values.

	Without regressing out mean FD			With regressing out mean FD		
	$F_{2, 104}$	*p* value	η_p^2	$F_{2, 104}$	*p* value	η_p^2
**Global properties of static FC network**						
Sigma	108.013	< 0.001	0.688	95.034	<0.001	0.660
Clustering coefficient	4.664	0.030	0.082	5.513	0.007	0.101
Global efficiency	6.193	0.005	0.112	3.624	0.038	0.069
Betweenness coefficient	6.007	0.005	0.109	114.321	<0.001	0.700
Modularity	182.344	< 0.001	0.788	161.508	<0.001	0.767
**Modular segregation index of static FC network**						
SOMH	31.741	< 0.0001	0.393	22.797	<0.001	0.318
VIS	54.490	< 0.0001	0.527	41.205	<0.001	0.457
CO	42.739	< 0.0001	0.466	37.560	<0.001	0.434
DM	60.849	< 0.0001	0.554	59.780	<0.001	0.550
MEM	36.995	< 0.0001	0.430	35.306	<0.001	0.419
VA	21.845	< 0.0001	0.308	22.820	<0.001	0.318
SAL	11.324	< 0.0001	0.188	9.037	<0.001	0.156
DA	4.010	0.025	0.076	4.248	0.017	0.080
SUB	33.879	< 0.0001	0.409	28.603	<0.001	0.369
**SD of dMSI (dynamic modular segregation index associated with dynamic FC network)**						
CON	5.837	0.004	0.106	5.761	0.004	0.105
VAN	7.501	0.001	0.133	5.650	0.005	0.103
**Speed of dMSI (dynamic modular segregation index associated with dynamic FC network)**						
CON	12.507	<0.001	0.203	11.558	<0.001	0.191
VAN	7.745	<0.001	0.136	5.702	0.005	0.104
FPN	5.779	0.004	0.105	6.006	0.005	0.109

SI Table 1. Main effects of cognitive states without or with controlling individual head motion differences in the statistical analysis. Significant results were reproducibly identified. SOMH: somatomotor hand, VIS: visual, CO: cingulo-opercular, DM: default mode, MEM: memory, VA: ventral attention, SAL: salience, FP: frontoparietal, DA: dorsal attention, SUB: subcortical. MSI, modular segregation index. SD, standard deviation. dMSI, dynamic modular segregation index.

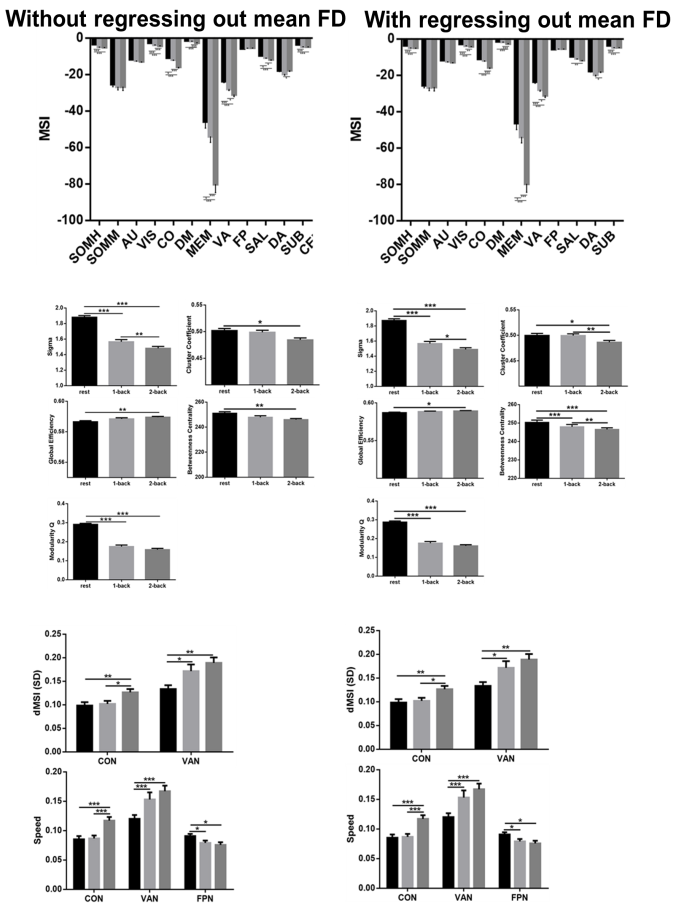

SI Figure 1. Post hoc comparison results of static and dynamic network reconfiguration without or with controlling individual head motion differences. Largely similar results were found without or with regressing out mean FD (left and right).

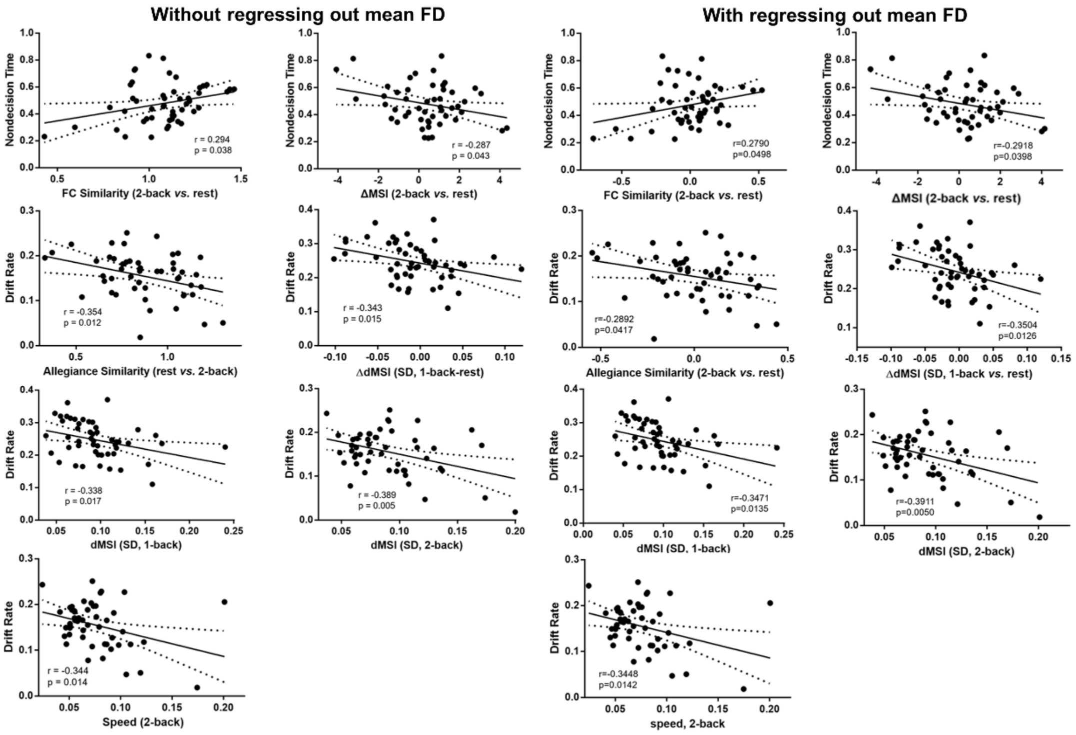

SI Figure 2. Similar correlation results were found between reconfiguration profiles and behavioral parameters after regressing out mean FD, suggesting the behavioral correlation was not likely to be attributed to individual head motion differences. MSI, modular segregation index of FPN. dMSI, dynamic modular segregation index of FPN. SD, standard deviation. FPN: frontoparietal network.

In addition to modularity maximization method, the consensus clustering method is another popular method to find a single solution for the nonconvex modularity maximization, which generates an agreement matrix for determining a representative network partition from multiple subjects or from a set of temporal dynamic networks (Bassett et al., 2013; Rasero et al., 2017; Kabbara et al., 2019), and increases the group separability (Rasero et al., 2019). To examine if our results could be affected by different method, we re-ran the consensus clustering (Lancichinetti and Fortunato. 2012). We first collected the results of 100 repetitions of Louvain modularity procedure (the same with modularity maximization method), and used the community affiliation vectors to calculate the agreement matrix (agreement.m from BCT). Then the agreement matrix was thresholded by 0.5 to remove elements of weak agreement (Conrad et al., 2020), and used for Louvain community detection with 100 repetitions, to evaluate potential differences between 100 partitions and generate new agreement matrix. This iterative process converged to a single representative partition that was the result of consensus clustering (consensus_und.m from BCT; Lancichinetti & Fortunato, 2012). Finally, the results of consensus clustering were compared with results of modularity maximization. As below the alluvial plot of consensus clustering showed similar pattern with our main results.

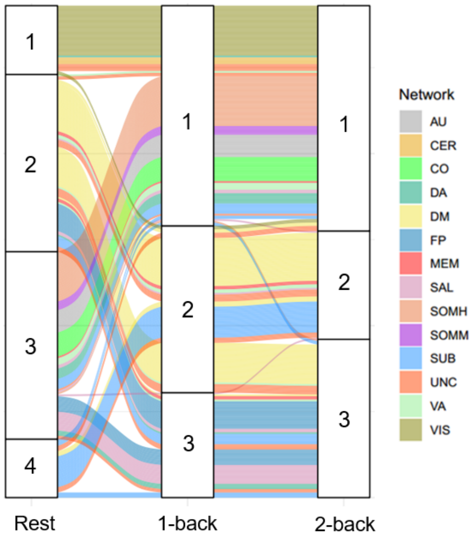

SI Figure 3. The alluvial "flow" demonstrated the modular structure reconfiguration in three cognitive states using consensus clustering method. Each streamline represents a node in the network, colored by the atlas' affiliation. The results showed that, from 1-back to 2-back task state, the modular structure was largely stable except some networks in module 2 and 3, like DM and SAL, were subdivided and switched to other modules. From rest to task state, module 3 separated a part to join in enlarged module 1, mainly involving SOMM, SOMH, AU, CO, DA, VA networks, while module 2 gave a part to module 3, mainly involving FP, SUB, and SAL networks. The different result was that, at rest the module 1 of "modularity maximization" seemed to be separated into module 2 and 4 of "consensus clustering". SOMH: somatomotor hand, SOMM: somatomotor mouth, AU: auditory, VIS: visual, CO: cingulo-opercular, DM: default mode, MEM: memory, VA: ventral attention, FP: frontoparietal, SAL: salience, DA: dorsal attention, SUB: subcortical, CER: cerebellum, UNC: uncertain.

To examine if our results were affected by different thresholds, we performed validation analyses for different thresholds (15%, 20%, and 25%). In total, our main results (20%) were largely reproducible at different thresholds, except several results (SI Table 2 and 3), were not significant but still showed similar trends modulated by cognitive loads at different thresholds (SI Figure 3 and 4).

	20% Threshold Main Results			15% Threshold			25% Threshold		
	Effect of cognitive state			Effect of cognitive state			Effect of cognitive state		
	$F_{2, 104}$	*p* value	η_p^2	$F_{2, 104}$	*p* value	η_p^2	$F_{2, 104}$	*p* value	η_p^2
**Global Topology**									
Sigma	108.013	< 0.001	0.688	94.343	< 0.001	0.658	112.810	< 0.001	0.697
Clustering coefficient	4.664	0.030	0.082	5.445	0.019	0.095	4.277	0.038	0.076
Global efficiency	6.193	0.005	0.112	10.366	< 0.001	0.175	2.845	0.076	0.055
Betweenness coefficient	6.007	0.005	0.109	9.633	< 0.001	0.164	2.796	0.079	0.054
**Modular Topology**									
MSI of SOMH	31.741	< 0.0001	0.393	35.235	< 0.0001	0.418	27.335	< 0.0001	0.358
MSI of VIS	54.490	< 0.0001	0.527	52.588	< 0.0001	0.518	57.338	< 0.0001	0.539
MSI of CO	42.739	< 0.0001	0.466	40.201	< 0.0001	0.451	44.067	< 0.0001	0.473
MSI of DM	60.849	< 0.0001	0.554	58.696	< 0.0001	0.545	59.161	< 0.0001	0.547
MSI of MEM	36.995	< 0.0001	0.430	23.607	< 0.0001	0.325	30.891	< 0.0001	0.387
MSI of VA	21.845	< 0.0001	0.308	18.977	< 0.0001	0.279	25.540	< 0.0001	0.343
MSI of SAL	11.324	< 0.0001	0.188	13.209	< 0.0001	0.212	11.002	< 0.0001	0.183
MSI of DA	4.010	0.025	0.076	——	n.s.	——	——	n.s.	——
MSI of SUB	33.879	< 0.0001	0.409	32.266	< 0.0001	0.397	32.702	< 0.0001	0.400

SI Table 2. Statistics of ANOVA demonstrated largely reproducible effect of cognitive state on functional network topology. The results of global efficiency and betweenness coefficient showed the trend ($P < 0.1$) at 25% threshold. SOMH: somatomotor hand, AU: auditory, VIS: visual, CO: cingulo-opercular, DM: default mode, MEM: memory, VA: ventral attention, SAL: salience, DA: dorsal attention, SUB: subcortical.

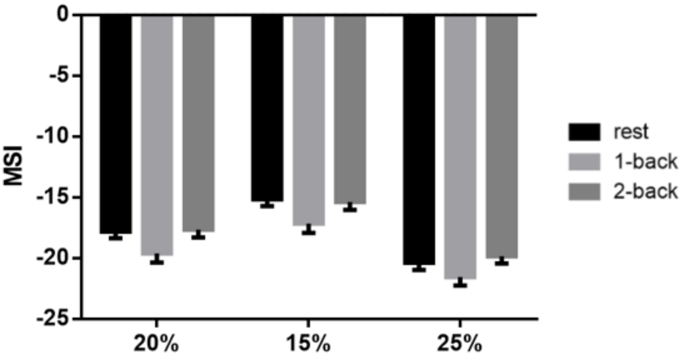

SI Figure 4. Statistics of ANOVA demonstrated similar trend of cognitive state effect on the MSI of DA at different thresholds (15%, 20%, and 25%).

	20% Threshold Main Results			15% Threshold			25% Threshold		
	Effect of cognitive state			Effect of cognitive state			Effect of cognitive state		
	$F_{2, 104}$	*p* value	η_p^2	$F_{2, 104}$	*p* value	η_p^2	$F_{2, 104}$	*p* value	η_p^2
**SD of dMSI**									
CO	5.837	0.004	0.106	10.888	<0.001	0.182	5.671	0.005	0.104
VA	7.501	0.001	0.133	——	n.s.	——	——	n.s.	——
**Speed of dMSI**									
CO	12.507	<0.001	0.203	15.233	<0.001	0.237	11.263	<0.001	0.187
VA	7.745	<0.001	0.136	5.088	0.010	0.094	5.844	0.004	0.107
FP	5.779	0.004	0.105	5.119	0.009	0.095	——	n.s.	——

SI Table 3. Statistics of ANOVA demonstrated moderately reproducible effect of cognitive state on the dynamic network topology such as the standard deviation (SD) and the speed of the dynamic modular segregation index (dMSI).

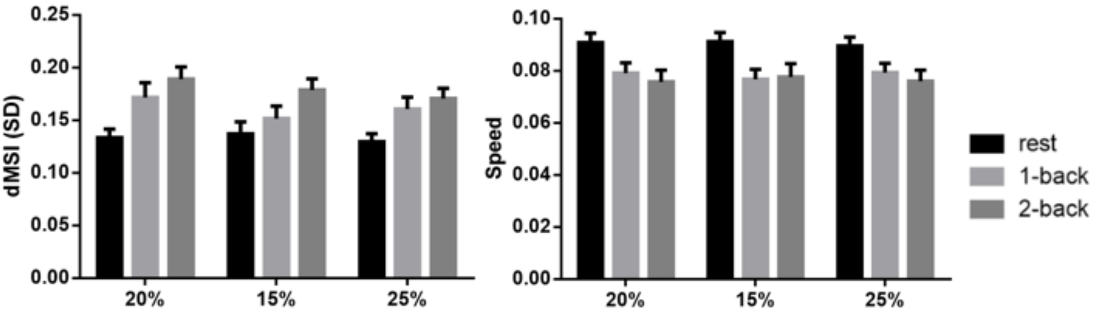

SI Figure 5. Statistics of ANOVA demonstrated similar trend of cognitive state effect on the SD of dMSI in VA (left) and speed of dMSI in FP (right) at different thresholds (15%, 20%, and 25%).

To examine if our results were affected by the choice of window lengths and step lengths, we performed validation analyses for dFC results i.e. standard deviation (SD) and speed of dMSI (SI Figure 5). The ANOVA and post hoc results with different combinations of window and step length displayed highly similar patterns, which reflected our results were reproducible. To delineate the trend of results with different window length and step length, we didn't use FDR correction but the liberal threshold of uncorrected $p < 0.05$.

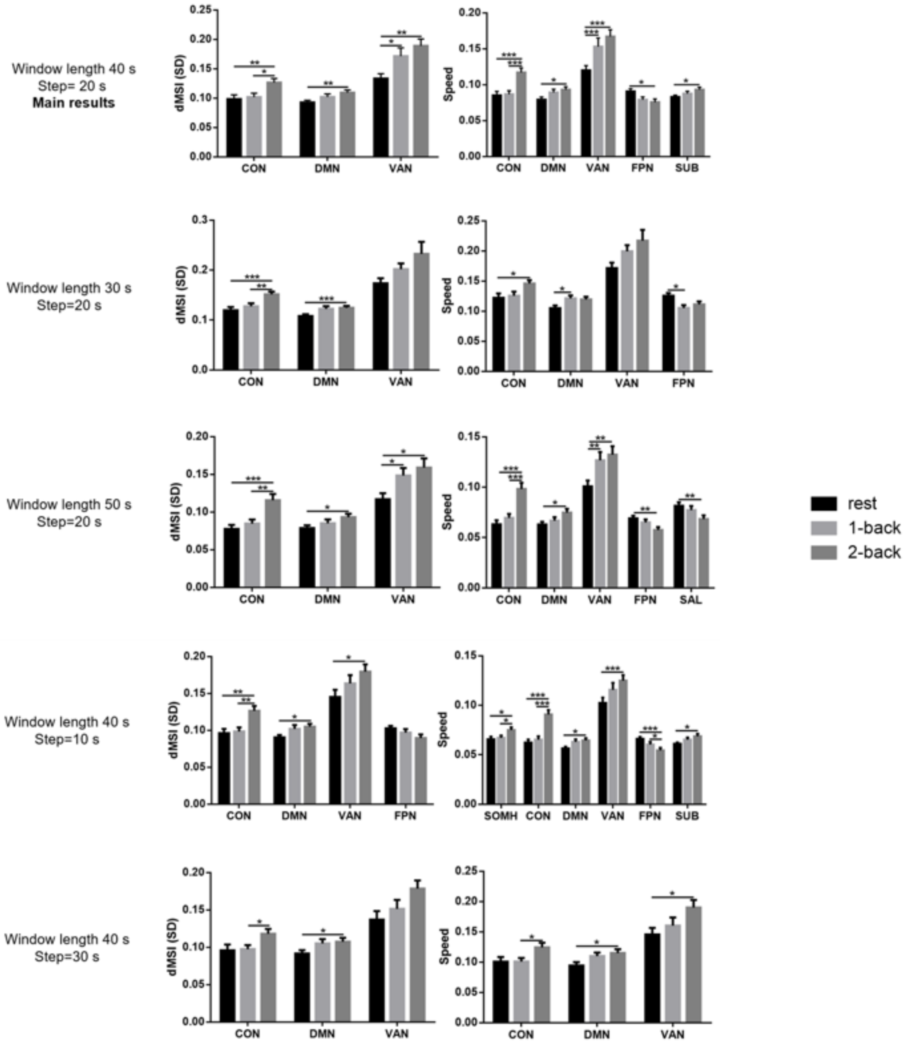

SI Figure 6. ANOVA and post hoc results of SD (first column) and speed (second column) of dMSI, with different window and step length. For the SD of dMSI, the effects of cognitive states in CON, DMN and VAN were reproducible. The trends of changes with increasing cognitive load were consistent too (first column). For the speed of dMSI, the effects of cognitive states were reproducible in CON, DMN, VAN, and moderately reproducible in FPN. Moreover, the trends of changes with increasing cognitive load were consistent too (second column). On the other hand, the SD of dMSI in FPN, and the speed of dMSI in SAL, SOMH and SUB seemed sensitive to window/step changes, particularly at the window length of 40 s and step of 10 s. SOMH: somatomotor hand, AU: auditory, CON: cingulo-opercular, DMN: default mode, VAN: ventral attention, FPN: frontoparietal, SAL: salience, SUB: subcortical. SD, standard deviation. dMSI, dynamic modular segregation index.

References

Bassett, D. S., Porter, M. A., Wymbs, N. F., Grafton, S. T., Carlson, J. M., & Mucha, P. J. (2013). Robust detection of dynamic community structure in networks. Chaos (Woodbury, N.Y.), 23(1), 013142.

Conrad, B. N., Wilkey, E. D., Yeo, D. J., & Price, G. R. (2020). Network topology of symbolic and nonsymbolic number comparison. Network neuroscience (Cambridge, Mass.), 4(3), 714–745. https://doi.org/10.1162/netn_a_00144

Kabbara, A., Khalil, M., O'Neill, G., Dujardin, K., El Traboulsi, Y., Wendling, F., & Hassan, M. (2019). Detecting modular brain states in rest and task. Network neuroscience (Cambridge, Mass.), 3(3), 878–901.

Lancichinetti, A., & Fortunato, S. (2012). Consensus clustering in complex networks. Scientific reports, 2, 336. https://doi.org/10.1038/srep00336

Rasero, J., Diez, I., Cortes, J. M., Marinazzo, D., & Stramaglia, S. (2019). Connectome sorting by consensus clustering increases separability in group neuroimaging studies. Network neuroscience (Cambridge, Mass.), 3(2), 325–343.

Rasero, J., Pellicoro, M., Angelini, L., Cortes, J. M., Marinazzo, D., & Stramaglia, S. (2017). Consensus clustering approach to group brain connectivity matrices. Network neuroscience (Cambridge, Mass.), 1(3), 242–253.
